# Soft and Hard Tissue Management in Implant Therapy—Part II: Prosthetic Concepts

**DOI:** 10.1155/2012/356817

**Published:** 2012-07-03

**Authors:** Paolo Francesco Manicone, Luca Raffaelli, Marjan Ghassemian, Antonio D'Addona

**Affiliations:** Institute of Clinical Dentistry, Catholic University of The Sacred Heart, Largo A. Gemelli 8, 00168 Rome, Italy

## Abstract

The ongoing pursuit of aesthetic excellence in the field of implant therapy has incorporated prosthetic concepts in the early treatment-planning phase, as well as the previously discussed surgical concepts. The literature has addressed these prosthetic and laboratory approaches required to enhance and perfect the soft and hard tissue management (SHTM). After surgically providing an acceptable hard tissue architecture and adequate timing of loading of the implant, the prosthetic phase is responsible for the soft tissue modeling, through correctly planned and executed procedures, which induce a satisfactory soft tissue profile by considering the microvasculature, the abutment connection and positioning, and the implementation of an adequate provisional phase. The objectives are the modeling of the soft tissues through the use of a conforming periorestorative interface which will produce desired and stable results.

## 1. Introduction

As a component of the key factors responsible for the aesthetic success of implant therapy, the prosthetic aspect is equally as important as the surgical aspect. The surgical aspect should provide a reliable hard tissue foundation for the creation of a harmonious soft tissue profile, which is completed and perfected by a well-planned and carried out prosthetic phase. It Commences with the functional loading of the implant, with an appropriately designed abutment and temporary restoration in a provisional phase of treatment, which is responsible for the soft tissue modeling, followed by the placement of the permanent restoration once the desired soft tissue form has been obtained, which effectively maintains the results achieved through long-term stabilization of the tissues. The fundamental concepts that will be discussed in this paper are the timing of the loading of the implant, the principles and techniques used for tissue modeling, and the management of the periorestorative components interface to achieve stabilization of adequate and predictable aesthetics.

## 2. Timing of Loading 

### 2.1. Timing Selection

The decision of when to load an implant is an important decision that is made by considering both the surgical and prosthetic aspects, which are responsible for the hard tissue integration to the implant and the final soft tissue results.

The timing of loading of an implant has been associated with the successful integration of an implant. Early studies of osseointegrated implants state that absence of micromovements and primary implant stability are important factors which contribute to the success of implant integration [1], as micromovements and reduced primary stability may lead to the formation of a soft tissue between the bony and implant surface. In addition, an excessive and uncontrolled mechanical load is thought to be a key factor that can contribute to implant failure due to bone remodeling, which may lead to increased or excessive marginal bone loss [2]. Conventional loading protocols require a healing period during which the implants placed are not to be functionally loaded. The rationale has been that by delaying the functional loading, micromovements would be reduced or eliminated and primary stability maximized, thus, allowing for proper osseointegration.

However, subsequently, the practice of immediate loading has been introduced, where implants are subjected to functional loading immediately or shortly following implant placement [3]. The obvious advantages of this protocol were the reduction in total treatment time and elimination of provisional removable prosthetics, but, subsequently, it was noticed that the other beneficial results were improved gingival aesthetics, which resulted from an earlier modeling of the soft tissues. Authors have concluded that in the case of the edentulous maxilla, the cervical contour of the immediate provisional prosthesis and its corresponding embrasures seem to induce the final shape of the peri-implant mucosa, during the healing phase [4], and in the case of the edentulous mandible the variability in the final result is found not to be dependant on the time of loading [5]. 

Studies comparing the conventional and immediately loaded protocols have been carried out by a number of authors to analyze the varying occlusal characteristics both in partially edentulous and edentulous subjects. The studies are in agreement and have reported that there are no significant differences seen between these protocols in terms of implant stability, marginal bone changes, and soft tissue response [6]. Therefore, researchers believe that implants can be successfully loaded immediately or shortly thereafter, so long as sufficient primary stability is achieved [7, 8], though it is believed that successful results may not be achieved by all clinicians [7].

The bone-to-implant contact is a histological and clinical characteristic that contributes to the stability of the implant within the bone. Primary bone-to-implant contact is defined as the amount of original bone that is in contact with the implant at the time of placement, and the secondary bone-to-implant contact is defined as the bone that is formed around the implant after-placement [9]. And results from an in vitro clinical trial on canine models comparing such histological, and other clinical and radiographic consequences of differently loaded single-tooth implants have indicated that no significant statistical differences were noted between these protocols [10].

However, a most recent study has examined the histological changes in socket healing in human subjects at various stages [11]. This research only partly corroborates with previous animal studies in terms of histological changes in the postextraction socket, demonstrating that only in the early stages the healing observed in canines was similar to that of humans. This research indicates that, in contrast to what was previously thought, hard tissue formation in humans is slower than that of the canine models, and the rate of new hard tissue formation varies greatly. Furthermore, this research indicated that the timing in which mineralized bone is deposited is not as predictable as originally thought and concluded that by the 24th week following extraction of the tooth, the structural organization of the bone was not completed. In light of these new results, and by applying these findings of socket healing to the healing that takes place following the placement of an implant, and contrarily to what previous studies indicated in relation to different loading protocols, the timing of loading of an implant must be carefully considered, and often a more delayed prosthetic functional loading may be the safer protocol to carry out. 

## 3. Soft Tissue Modeling

There are both anatomic and prosthetic factors that are responsible for a well-accomplished modeling of the soft tissues. Firstly, an adequate blood supply must be present to ensure the responsiveness of the soft tissues to the prosthetic stimuli, followed by accurately connected and positioned abutment, which becomes the foundation for a well-designed provisional prosthetic component.

### 3.1. Microvascular Function

**Figure 1:**
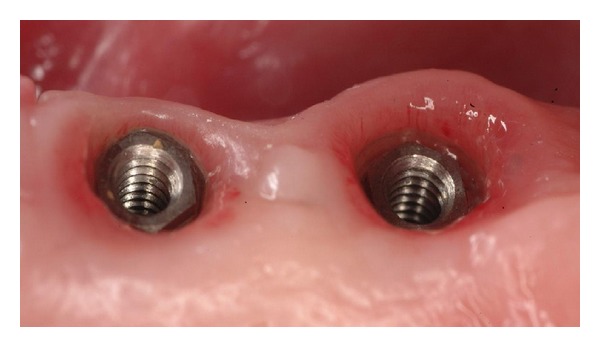
Intraoral view of healthy peri-implant soft tissues, with visible microvessels.

Earlier studies of the microcirculation in rat subjects [12] have indicated that the mucosal microvasculature gingival tissues adjacent to implants are similar to those of natural teeth. From this it can be deduced that an adequate bloody supply to the peri-implant tissues is necessary for the nutritional requirements of the gingival tissues, just as it is for the gingival tissues around natural teeth (Figure 1).

The maintenance of an adequate blood supply of the peri-implant tissues is an important factor both during the surgical phase by preventing the undesired effects such as the loss of tissues following the surgical procedures and for the nutritional requirements of the tissues during the tissue conditioning stage of the prosthetic phase, either by the healing abutment or by the provisional restoration [13]. A reduced blood supply may be the result of the conventional surgical implant placement protocol or may be caused by increased pressure of the prosthetic components, which may lead to ischemia of the tissues resulting in necrosis or recession of the gingival tissues. 

### 3.2. Abutment Connection and Positioning

Currently, there are many types of abutment connections, depending on the type of final restoration and type of implant. The implant-abutment interface connections can have either internal or external connections, and extensive research of its geometry has been carried out because it is crucial and consequential to prosthetic stability [14]. In addition, particular attention has been given to this region because research has suggested that bacterial colonization and plaque formation, which occur at the microgap of this interface, may cause tissue inflammation and bone resorption in the dog model [15], as a consequence, the concept of platform switching has been proposed to preserve bone around the head of a wide-diameter implant [16]. 

Another aspect of the abutment connection, which has been studied and thought to have an effect upon the soft tissue architecture, is the repetition of abutment removal and subsequent reconnection. Findings from this research confirmed previous data describing the transmucosal barrier, which is made up by an epithelial and connective tissue layer [17–19]. In addition, it was found that frequent dis- and connections of the abutment compromised the integrity of the mucosa and caused tissue reactions which resulted in a more apically placed surface of connective tissue integration and in bone resorption, via mechanical disruption of the established biological width [20].

Subsequently, even the tissue response following the replacement of the healing abutment for a permanent abutment was researched. Results from a study carried out in canine models analyzing the tissue reactions that occurred following the removal of a healing abutment and placement of a permanent abutment indicated that this replacement did not result in further bone loss and thus, a single abutment shift did not jeopardize the mucosal attachment, as the connective tissue attachment to the titanium abutment was reestablished [21].

Although there are different timing protocols for the positioning of abutments, current literature recommends that provisional restorations be fabricated by making silicone matrices from the diagnostic wax ups prior to commencing the surgical procedures which can be relined intraorally and refined in the laboratory [22]. These provisional crowns are usually placed upon temporary abutments and maintained in the tissue conditioning phase. 

### 3.3. Provisional Phase 

A thorough understanding of the histological aspects of the healing phase also contributes to the success of the provisional phase, in promoting maintenance of health and soft tissue modeling, through correct contour, marginal fit, and interproximal spacing of the temporary crown [23] to establish the composition of the surface between the prosthetic recipient site and the restorative gingival interface [24]. In addition to this, extensive animal research has been carried out to examine the histological characteristics of soft tissue maturation stage following the placement of implants. Furthermore, it has been determined in the dog model that histological similarities exist between the initial stages of healing after extraction, and healing of soft tissues after placement of an implant. In the postextraction socket, the final stages of healing consist mainly of osteoid formation in the socket with epithelium formation to cover the socket, whereas the final stages of healing of peri-implant soft tissue comprise mainly formation and maturation of epithelial tissue, which take place within 6–8 weeks [25]. Keeping in mind these concepts, guided gingival regeneration can be used in the exposure and healing phase of placement of the healing abutment to develop a correct gingival contour [26].

Buser et al., in 1992 [27], described in the dog model the connective tissue component of the peri-implant tissues as an inflammation-free scar tissue. This indicates that the morphogenesis of these tissues are a dynamic process, which can be guided or modified. Therefore, the selection of an adequately designed provisional restoration in single-implant cases, substantially affects the aesthetic result during this phase of treatment [28].

Although the main goals of the provisional phase remain unchanged, over the years, different variations of techniques using provisional restorations of different materials during the phase of treatment for developing an aesthetic gingival peri-implant contour have been used, including the cervical contouring concept [29]. 

In our experience, to minimize abutment dis/and reconnection and maximize aesthetic soft tissue modeling, one of the techniques which has been employed for submerged and ideally positioned implants involves the following steps [22, 28–30]: (1) taking the final impression at the uncovering stage with a minimal flap access; (2) casting of the definitive abutment, framework, and provisional crown with anatomical characteristics derived from an ideal wax up; (3) immediate placement of the definitive abutment and provisional crown after 24 hours, allowing the healing of the soft tissue around an ideal profile and form of restoration; (4) when ideal architecture of the soft tissue is obtained, and after the removal of the provisional crown and positioning of the framework, an impression for the soft tissue profile is taken; (5) for the laboratory procedures, by using a silicon index of the initial wax up, the ceramic layering of the framework is realized with the same design as the provisional crown; (6) a final restoration identical to the initial wax up and the definitive crown can be positioned without altering the soft tissue stability enhanced during the healing phase.

## 4. Soft Tissue Stabilization 

As much as all of the abovementioned concepts play a key role in the modeling of the peri-implant gingival tissues, arguably, the most significant incentive to favorable gingival aesthetics is given by an appropriately selected abutment with an anatomically designed final restoration. The significant factors which should generate and contribute to an optimal aesthetic result include the abutment selection, the crown design, and the soft tissue outcome.

### 4.1. Abutment Selection 

The principles and guidelines for complete tooth preparations for fixed prosthesis have been reviewed and discussed in the literature and include the occlusal convergence, occlusocervical dimension, circumferential morphology, finish line location, form and depth, and others. All these principles are thought to produce a tooth preparation which is sound mechanically, biologically, and aesthetically and have been thoroughly discussed [31]. In addition, authors believe that by associating the principles of ideal tooth preparations with precise tooth form dimensions, exemplary machine-milled abutments can be generated [32]. 

More specifically to implant supported restorations, however, the most important ideal characteristics for the selection, fabrication, and placement of implant abutments have been described and include an exact interface between the abutment and implant, having the possibility to modify the long axis of the implant with an angled abutment and using an abutment that fits passively with a correct emergence profile and a finishing line that follows the morphology of the peri-implant tissues [30].

Permanent abutment connections can be classified in many ways, because implants have been used in a variety of reconstructive procedures, ranging from single-tooth implant to implant retained full prosthesis, and to be able to handle different clinical situations [14]. Whichever the type of abutment that is used, the fabrication techniques used to date are stock abutments which can be modified in the chair, cast-metal abutments, and CADCAM-milled abutments. 

Though ideal aesthetics of implant therapy begin with correctly placed and positioned implants: however, implants with jeopardized positions should be rectified with modified abutments [33]. Recently, these different abutment-fabrication methods have been compared to identify the most advantageous method of fabrication. From the literature, advantages of using modified or customized abutments over stock abutments have been reported [34].

To further classify and identify the best characteristics of customized abutments, the techniques used for customized abutment fabrication are classified into a casting of the wax pattern, into ceramic or gold abutments, machine-milled titanium abutments, or customizable prefabricated titanium abutments [32]. Some of the advantages of customized abutments include changes in direction and position of the axis of the final restoration and modification of the form, diameter and gingival margin position, which induce better aesthetics.

As well as differentiating between differently designed abutments, several authors have studied the effects on soft tissue by using implant components of different materials. Other than the original titanium components, the other materials which have been studied extensively include zirconia and a gold alloy. In vitro studies analyzing the biological properties of zirconia have indicated that it has low cytotoxic properties and strongly induces adhesion of fibroblasts to its surface, by increasing the cellular growth rate, as compared to feldspathic ceramics [35]. Other clinical data published to date suggest that restorations made of zirconium oxide are adequately tolerated and resistant [36], resulting in favourable mucosal conditions and marginal bone levels [37]. 

An animal study indicates that the soft tissue healing of both titanium and zirconia abutments remain, stable between 2 and 5 months, whereas a gold/palladium alloy abutments showed signs of “apical shift of the barrier epithelium and marginal bone between 2 and 5 months of healing,” which may result from lower amounts of collagen and fibroblast and greater amounts of leukocytes than in titanium and zirconia abutments [38]. These results are in contrast with a recent systematic review of the literature that has analyzed the difference in peri-implant stability of titanium abutments, compared with gold, aluminum, and zirconium oxide [39]. The conclusions collected by the authors indicate that there is no evidence to prove that titanium abutments perform better in terms of maintaining an unaltered tissue condition as compared to the other materials. Another randomized controlled clinical study comparing the technical and biological characteristics of titanium and zirconia abutments such as probing pocket depths, plaque control records, bleeding on probing, bone level measurements, and difference in colour of the periodontal tissues at 6, 12, and 36 months found that both materials exhibited similar properties [40].

Another animal study found similar bone and soft tissue dimensions when using either titanium or gold transmucosal parts. It is thought that these differences may be due to different methodologies. This study seems to confirm other previous studies in demonstrating that the attachment and proliferation of the epithelial cells in the metallic surfaces are favourable especially on smooth surfaces [41]. 

Another characteristic of the abutment which has been recently analyzed is the surface topography and results from this systematic review indicate that rougher surfaces promote the formation of plaque [42]. 

## 5. Conclusions

Considering the increase in demand for better aesthetics in recent years, there has been a change in the treatment planning and execution of the clinical and laboratory procedures of implant-supported restorations. The realization requires collaboration between operators, including the surgeon, the prosthodontist, and the dental technician, to obtain a predictable, satisfactory and maintainable aesthetic result. From a prosthetic aspect, the optimization of the SHTM requires an accurate planning of prosthetic paradigms: an adequate timing of loading, and the selection of an ideal abutment that is capable of guiding the remodeling of the soft tissues during the provisional phase, allowing a thorough stabilization and integration of the definitive restoration with the remaining natural dentition.
